# Optimal Adjuvant Therapy Selection for Chinese BRAF V600‐Mutant Stage III Melanoma: A Multicenter Efficacy Comparison of Targeted Agents, Immunotherapy, and Combinatorial Strategies

**DOI:** 10.1002/mco2.70738

**Published:** 2026-04-25

**Authors:** Rongcheng Zhang, Yao Liang, Jingjing Li, Qianqi Chen, Ya Ding, Xizhi Wen, Baiwei Zhao, Wei Zheng, Junwan Wu, Qiong Zhang, Ziluan Chen, Qiuyue Ding, Linbin Chen, Renai Li, Ke Li, Qiming Zhou, Xiaoshi Zhang, Dandan Li

**Affiliations:** ^1^ Biotherapy Center State Key Laboratory of Oncology in South China Guangdong Provincial Clinical Research Center for Cancer Collaborative Innovation Center for Cancer Medicine Sun Yat‐sen University Cancer Center Guangzhou People's Republic of China; ^2^ State Key Laboratory of Oncology in South China Collaborative Innovation Center for Cancer Medicine Sun Yat‐Sen University Cancer Center Guangzhou People's Republic of China; ^3^ Department of Gastric Surgery Sun Yat‐sen University Cancer Center Guangzhou People's Republic of China; ^4^ Department of Oncology Huazhong University of Science and Technology Union Shenzhen Hospital Shenzhen People's Republic of China; ^5^ State Key Laboratory of Oncology in South China Guangdong Provincial Clinical Research Center for Cancer Sun Yat‐sen University Cancer Center Guangzhou People's Republic of China; ^6^ Department of Ultrasound State Key Laboratory of Oncology in South China Collaborative Innovation Center for Cancer Medicine Sun Yat‐Sen University Cancer Center Guangzhou People's Republic of China; ^7^ Department of Cancer Biotherapy Center Cancer Center of Yunnan Province Yunnan Cancer Hospital The Third Affiliated Hospital of Kunming Medical University Kunming People's Republic of China

**Keywords:** adjuvant therapy, BRAF V600 mutation, melanoma, targeted therapy, target‐immune combination therapy

## Abstract

While adjuvant immunotherapy and BRAF/MEK inhibitors improve the outcomes for BRAF V600‐mutant stage III melanoma, comparisons of long‐term survival and safety of these therapeutic modalities are currently lacking in Chinese patients. We retrospectively analyzed data from patients with resected stage III BRAF V600‐mutant melanoma who received adjuvant therapy between June 2013 and December 2023 across three centers in China. Note that 122 patients were included and categorized into interferon (*n* = 25), aPD‐1 (*n* = 18), D/T (*n* = 62), and BRAFi/aPD‐1 (*n* = 17) cohorts. The D/T group demonstrated a significantly longer median RFS compared to the interferon and aPD‐1group (22.7 vs. 11.9 months, *p* = 0.005; vs. 12.5 months, *p* < 0.001). Similar results were obtained by restricted‐mean‐survival‐time model. Patients who continued D/T beyond 1 year exhibited significantly improved RFS and DMFS compared to those who discontinued at 1 year duration (NR vs. 22.0 months, *p* = 0.048; NR vs. 22.5 months, *p* = 0.026). NOTCH4 and IL7R mutations may serve as prognostic and predictive biomarkers for long‐term survival and targeted‐immunotherapy efficacy, respectively. Adjuvant therapy with D/T may represent the most effective treatment strategy for Chinese patients with stage III melanoma harboring BRAF V600 mutations. A combination of BRAF‐targeted therapy and aPD‐1 immunotherapy provided comparable efficacy and may be an alternative for a specific patient.

## Graphical abstract

Adjuvant dabrafenib plus trametinib (D/T) therapy appears to offer the greatest clinical benefit for Chinese patients with stage III BRAF V600‐mutant melanoma. Furthermore, to maximize therapeutic efficacy in this population, extending D/T therapy beyond 1 year may be advisable. For Chinese patients who are unable to tolerate the febrile adverse events of D/T or have contraindications to MEK inhibitors due to ocular or cardiac comorbidities, BRAFi combined with aPD‐1 may serve as a feasible alternative.

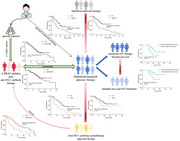

## 1 Introduction

Melanoma originates from melanocytes and is responsible for the majority of skin cancer‐related deaths despite its lower incidence compared to other cutaneous malignancies [1, 2]. BRAF mutations are commonly observed in melanoma, with a reported mutation rate of 25.5% among Chinese patients diagnosed with this condition [3]. Compared to wild‐type melanoma, BRAF‐mutant cases tend to have an earlier onset, a more aggressive disease progression, and an increased likelihood of being identified at an advanced stage [4]. In the absence of adjuvant therapy, approximately half of patients with resected stage III BRAF‐mutant melanoma are likely to experience disease recurrence within 1‐year following surgical intervention, and 23% may succumb to the illness within 3 years [5]. These findings highlight the necessity for adjuvant therapy in patients with stage III BRAF‐mutant melanoma post‐surgery.

High‐dose interferon (HDI) was the inaugural adjuvant therapy sanctioned for stage III melanoma following surgical resection [6]. In the last decade, the U.S. Food and Drug Administration (FDA) has approved several novel therapies for melanoma, particularly the advent of immunotherapy and BRAF/MEK inhibitors, which have significantly improved the prognosis for patients undergoing adjuvant treatment [7, 8, 9, 10]. Despite these advances, the optimal selection among available adjuvant strategies remains clinically challenging, as treatment decisions must balance efficacy, toxicity profiles, treatment adherence, and patterns of disease recurrence.

Chinese melanoma patients present distinct clinical and molecular characteristics compared with Western cohorts. These include a lower tumor mutational burden (TMB), a higher prevalence of structural genomic alterations, and a different distribution of melanoma subtypes, such as a higher proportion of acral and mucosal melanoma [11, 12, 13]. Such differences may influence response to immunotherapy and limit the direct generalizability of findings from Western clinical trials [12, 14]. In addition, there is a paucity of large‐scale clinical trial data on BRAF/MEK inhibitors in the adjuvant setting for Chinese patients. Real‐world factors, including treatment accessibility, adherence, and heterogeneity in clinical practice, may further contribute to variations in outcomes.

In light of these considerations, we conducted a multicenter, retrospective cohort study aimed at evaluating the long‐term survival benefits and safety profiles associated with the most commonly utilized adjuvant treatment strategies in Chinese patients with resected stage III BRAF V600‐mutant melanoma over the past decade.

## 2 Results

### 2.1 Patients

**FIGURE 1:**
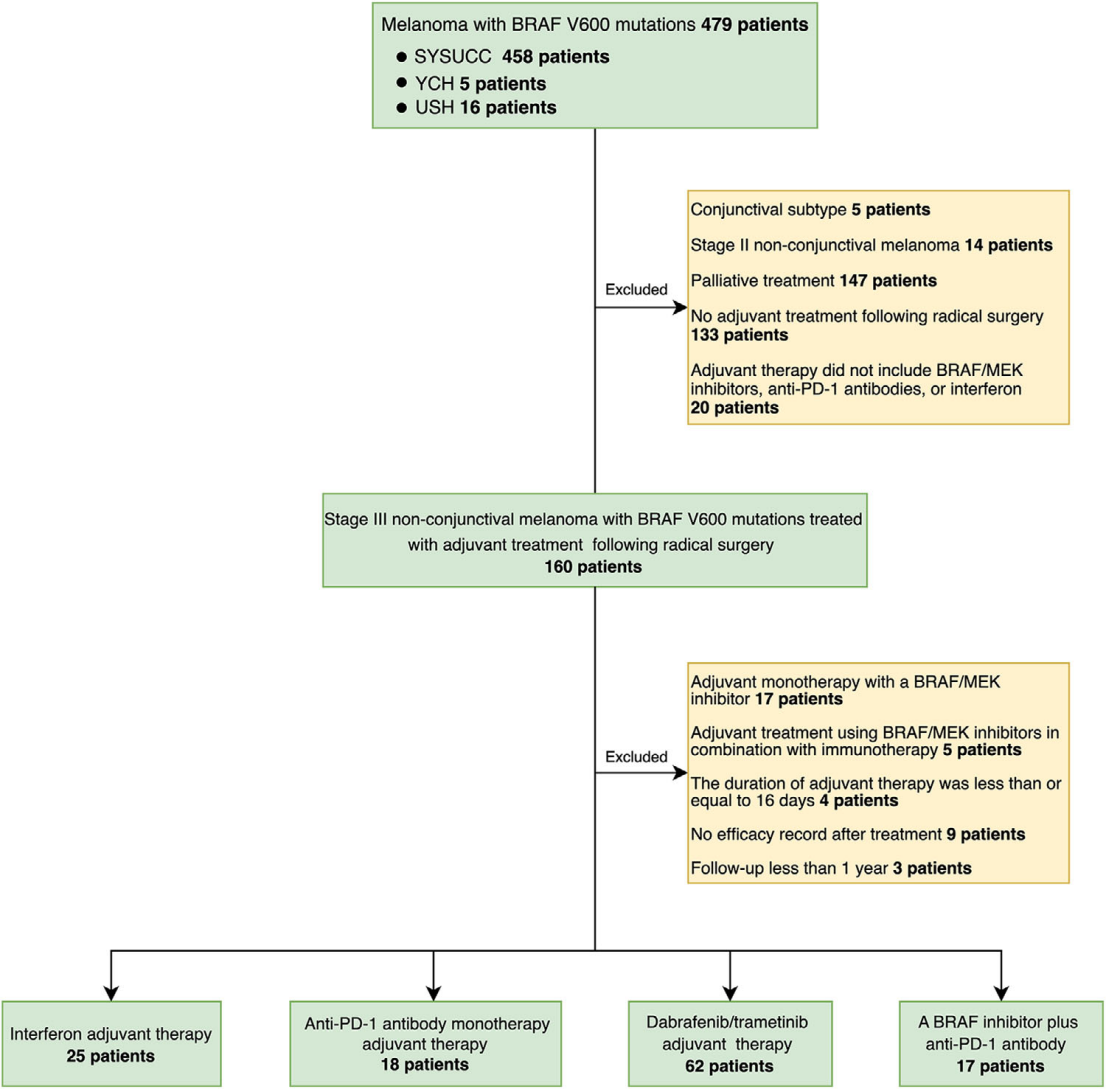
Flow diagram of patient screening. SYSUCC, Sun Yat‐sen University Cancer Center; USH, Union Shenzhen Hospital; YCH, Yunnan Cancer Hospital.

A total of 479 melanoma patients with BRAF V600 mutations were identified, from which 122 patients with stage III melanoma who had undergone surgical resection were ultimately included in the study (Figure 1). These patients received adjuvant therapy between June 2013 and December 2023, which comprised interferon (*n* = 25, 20%), anti‐PD‐1 therapy (*n* = 18, 15%), dual therapy (D/T) (*n* = 62, 51%), and a combination of BRAF inhibitors and anti‐PD‐1 therapy (BRAFi/aPD‐1) (*n* = 17, 14%). At the initiation of treatment, the baseline characteristics of the patient groups were generally comparable (Table 1). The observed variations in BRAF V600 mutation subtypes and baseline serum amyloid A (SAA) levels were primarily attributable to the absence of relevant testing in certain patients at the onset of treatment, leading to undetermined BRAF V600 mutation status and SAA levels.

**TABLE 1:** Patient characteristics.

Characteristic	Interferon group	aPD‐1 group	D/T group	BRAFi/aPD‐1 group	*p* value
	(*n* = 25)	(*n* = 18)	(*n* = 62)	(*n* = 17)	
Age, years (*n*, %)					0.5^a^
Median	48	48	48	48	
IQR	(33–57)	(35–59)	(37–56)	(39–59)	
< 60	21 (84.0)	13 (72.2)	52 (83.9)	15 (88.2)	
≥ 60	4 (16.0)	5 (27.8)	10 (16.1)	2 (11.8)	
Sex					0.3^b^
Male	15 (60.0)	7 (38.9)	24 (38.7)	6 (35.3)	
Female	10 (40.0)	11 (61.1)	38 (61.3)	11 (64.7)	
Melanoma subtype (*n*, %)					0.7^c^
Non‐acral cutaneous	18 (72.0)	14 (77.8)	50 (80.6)	14 (82.4)	
Acral	6 (24.0)	3 (16.7)	6 (9.7)	2 (11.8)	
Mucosal	0 (0)	0 (0)	4 (6.5)	1 (5.9)	
Unknown primary	1 (4.0)	1 (5.6)	2 (3.2)	0 (0)	
Stage (AJCC eighth) of patients with cutaneous and acral melanoma (*n*, %)					0.7^c^
IIIA	1 (4.0)	0 (0)	1 (1.6)	0 (0)	
IIIB	3 (8.0)	2 (11.1)	7 (11.3)	3 (17.6)	
IIIC	16 (64.0)	9 (50.0)	25 (40.3)	9 (52.9)	
IIID	0 (0)	0 (0)	5 (8.1)	1 (0)	
III unspecified	4 (16.0)	6 (33.3)	18 (29.0)	3 (17.6)	
BRAF V600E/K mutation (*n*, %)					< 0.01^c^
V600E	9 (36.0)	13 (72.2)	58 (93.5)	8 (47.1)	
V600K	1 (4.0)	0 (0)	1 (1.6)	2 (11.8)	
V600E/K	15 (60.0)	5 (27.8)	3 (4.8)	7 (41.2)	
SAA Level (*n*, %)					0.02^b^
Elevated	5 (20.0)	3 (16.7)	21 (33.9)	3 (17.6)	
Normal	6 (24.0)	8 (44.4)	20 (32.3)	12 (70.6)	
Unknown	14 (56.0)	7 (38.9)	21 (33.9)	2 (11.8)	

^a^ Kruskal–Wallis *H*.

^b^ Chi‐square test.

^c^ Fisher–Freeman–Halton exact test.

### 2.2 Efficacy

**FIGURE 2:**
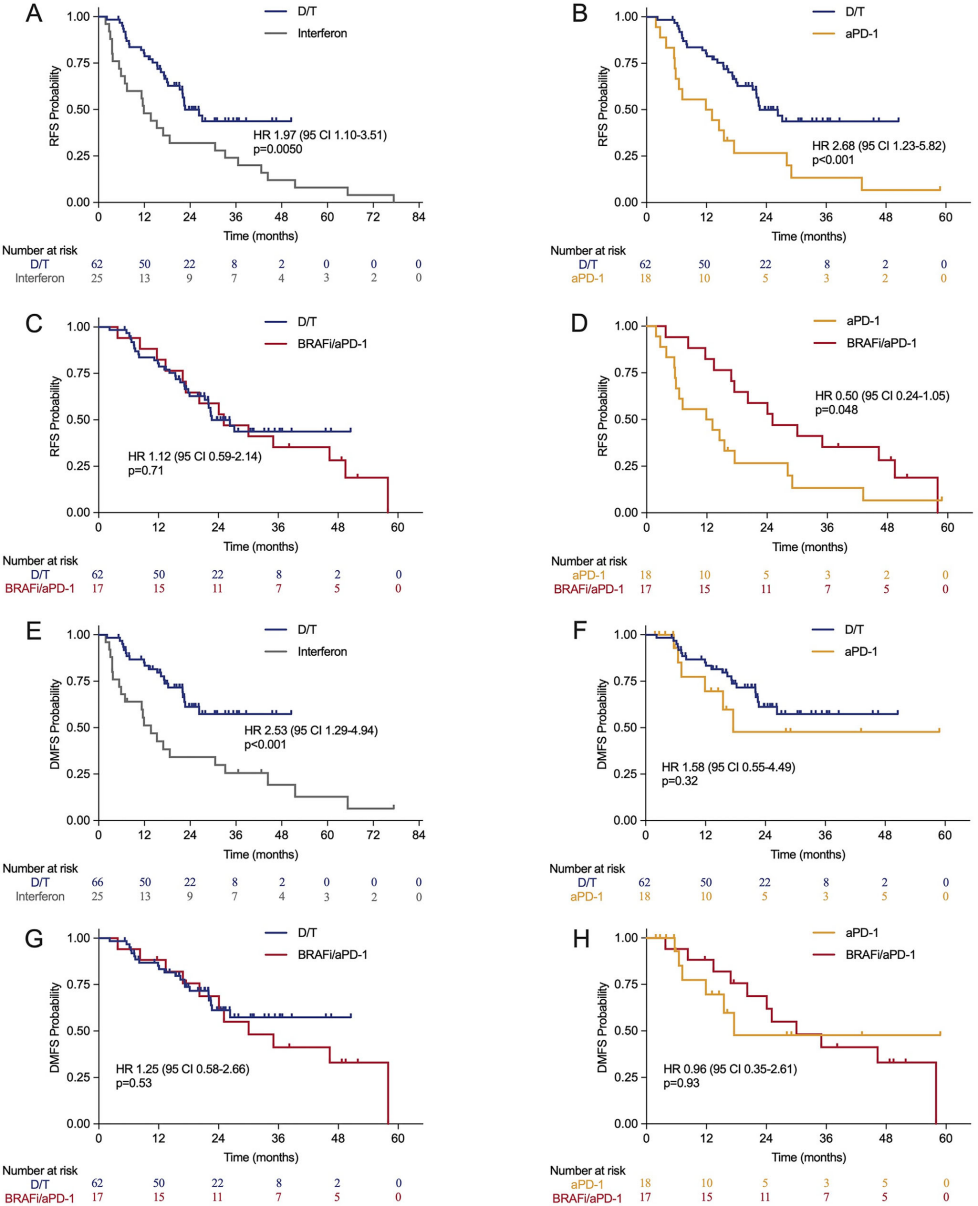
Survival outcomes of adjuvant interferon, aPD‐1, D/T, and BRAFi/aPD‐1‐treated patients with resected BRAF V600 mutant melanoma. (A) RFS between D/T and interferon (*p* = 0.005). (B) RFS between D/T and aPD‐1 (*p* < 0.001). (C) RFS between D/T and BRAFi/aPD‐1 (*p* = 0.71). (D) RFS between aPD‐1 and BRAFi/aPD‐1 (*p* = 0.048). (E) DMFS between D/T and interferon (*p* < 0.001). (F) DMFS between D/T and aPD‐1 (*p* = 0.32). (G) DMFS between D/T and BRAFi/aPD‐1 (*p* = 0.53). (H) DMFS between aPD‐1 and BRAFi/aPD‐1 (*p* = 0.93). aPD‐1, anti‐program death‐1 antibody; BRAFi, BRAF inhibitor; DMFS, distant metastasis‐free survival; D/T, dabrafenib/trametinib; RFS, relapse‐free survival.

The median follow‐up period was 21 months, with an interquartile range (IQR) of 11–34 months. The specific follow‐up durations for the interferon, aPD‐1, D/T, and BRAFi/aPD‐1 groups were 14, 13, 23, and 25 months, respectively. The relapse‐free survival (RFS) for the D/T group was significantly better than that of the interferon group and the aPD‐1 group, with median RFS values of 22.7 months compared to 11.9 months (*p* = 0.005) and 12.5 months (*p* < 0.001), respectively (Figure 2A,B). However, the RFS for the D/T group was marginally shorter than that of the BRAFi/aPD‐1 group, which had a median of 25.1 months (95% CI 16.9‐not reached [NR], *p* = 0.71) (Figure 2C). In terms of distant metastasis‐free survival (DMFS), the D/T group (NR, 95% CI 22.7‐NR) had a significantly longer DMFS than the interferon group (13.7 months, 95% CI 6.9‐44.4, *p* < 0.001), but did not differ significantly from the aPD‐1 group (17.5 months, 95% CI 16.9‐NR, *p* = 0.32) or the BRAFi/aPD‐1 group (30.0 months, 95% CI 16.9‐NR, *p* = 0.53) (Figure 2E–G). Both the D/T and BRAFi/aPD‐1 groups showed favorable rates for RFS and DMFS (Table 2). Although the RFS rates for the aPD‐1 group were inferior to those of the D/T group, the DMFS rates were comparable, particularly after a 2‐year period. Additionally, a direct comparison between the aPD‐1 group and BRAFi/aPD‐1 groups revealed a significant RFS advantage for the BRAFi/aPD‐1 group (*p* = 0.048), while no significant difference in DMFS was observed between the two groups (*p* = 0.93) (Figure 2D,H). No significant differences in overall survival (OS) were observed among the four adjuvant therapy groups (all *p* > 0.05) (Figure S1).

**TABLE 2:** RFS and DMFS by different adjuvant therapies.

	Interferon	aPD‐1	D/T	BRAFi/PD‐1
	(*n* = 25)	(*n* = 18)	(*n* = 62)	(*n* = 17)
**RFS**		
**Median (months, 95%CI)**	11.9 (6.9–33.2)	12.5 (5.8–29.0)	22.7 (21.3–NR)	25.1 (16.9–NR)
1‐year rate (%, 95%CI)	48.0 (31.9–72.2)	50.0 (31.5–79.4)	80.3 (71.0–91.0)	76.5 (58.7–99.5)
2‐year rate (%, 95%CI)	32.0 (18.1–56.7)	26.7 (12.1–58.6)	49.9 (38.1–65.4)	58.8 (39.5–87.6)
3‐year rate (%, 95%CI)	24.0 (12.0–48.2)	13.3 (3.8–46.9)	43.7 (31.5–60.6)	35.3 (18.5–67.2)
*p*‐value	0.005	< 0.001		0.71
**DMFS**		
**Median (months, 95%CI)**	13.7 (6.9–44.4)	17.5 (11.9–NR)	NR (22.7–NR)	30.0 (20.2–NR)
1‐year rate (%, 95%CI)	51.2 (34.7–75.5)	69.6 (48.7–99.6)	85.1 (76.5–94.6)	81.9 (65.3–100.0)
2‐year rate (%, 95%CI)	34.1 (19.6–59.6)	47.8 (25.1–90.7)	61.2 (48.7–76.8)	68.3 (48.6–95.9)
3‐year rate (%, 95%CI)	25.6 (12.9–50.8)	47.8 (25.1–90.7)	57.3 (44.2–74.4)	41.0 (22.3–75.3)
*p*‐value	<0.001	0.32		0.53

*Note*: aPD‐1, anti‐program death‐1 antibody; BRAFi, BRAF inhibitor; DMFS, distant metastasis‐free survival; D/T, dabrafenib/trametinib; NR, not reached; RFS, relapse‐free survival.

Univariable and multivariable Cox regression analyses conducted for RFS and DMFS identified adjuvant therapy as the only independent prognostic factor, with no other variables demonstrating statistically significant associations (all *p* > 0.05). Similarly, univariable analysis for OS revealed no significant correlations between clinicopathological variables and survival outcomes (all *p* > 0.3). Detailed results of the Cox regression analyses are presented in Tables S2–S4. To further corroborate these findings, restricted mean survival time (RMST) analysis at the 2‐year landmark confirmed consistent patterns, with both the D/T and BRAFi/aPD‐1 groups exhibiting longer RMST for RFS and DMFS compared to other treatment regimens. No significant differences in RMST for OS were noted (all *p* > 0.05). Comprehensive 2‐year RMST estimates across cohorts are summarized in Table S5.

**FIGURE 3:**
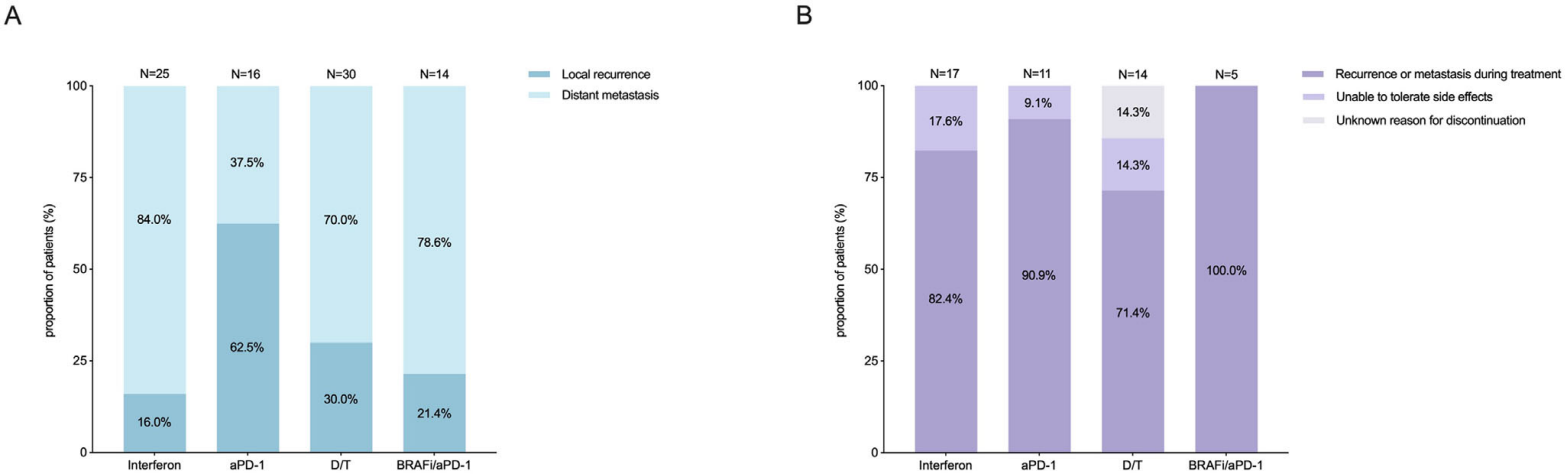
Recurrence patterns among the four adjuvant treatment groups and reasons for less than 1 year of treatment. (A) At the time of follow‐up, the proportion of local recurrence and distant metastasis among patients who experienced recurrence in each group. (B) Specific causes leading to patients treated for less than 1 year in each group. aPD‐1, anti‐program death‐1 antibody; BRAFi, BRAF inhibitor; D/T, dabrafenib/trametinib.

At the time of the last follow‐up, disease recurrence or metastasis was documented in 25, 16, 30, and 14 patients in the interferon, aPD‐1, D/T, and BRAFi/aPD‐1 groups, respectively, with median recurrence times of 11.9 months (95% CI 6.9–33.2), 9.5 months (95% CI 5.6–28.1), 14.8 months (95% CI 11.2–18.2), and 22.1 months (95% CI 15.5–49.5). The aPD‐1 group predominantly exhibited local recurrence (62.5%), while distant metastasis was the primary recurrence pattern in the other three groups (Figure 3A). In patients who developed distant metastases, the distribution of metastatic sites varied across treatment groups (Figure S3). Subcutaneous nodules were most frequently observed in the interferon group. Liver metastases were most commonly observed in patients treated with aPD‐1. Brain metastases were predominant in the D/T group, while lung metastases were the most prevalent in the cohort treated with the BRAFi/aPD‐1 combination. In the aPD‐1 group, the median time to local recurrence was 9.5 months (95% CI 3.93‐NA). In the BRAFi/aPD‐1 group, all patients received a BRAFi lead‐in prior to aPD‐1 initiation, with a median interval of 1.6 months (95% CI 1.1–4.4) between treatments. No patients experienced rapid disease progression during the BRAFi lead‐in period, and all proceeded to receive BRAFi plus aPD‐1 combination therapy.

**FIGURE 4:**
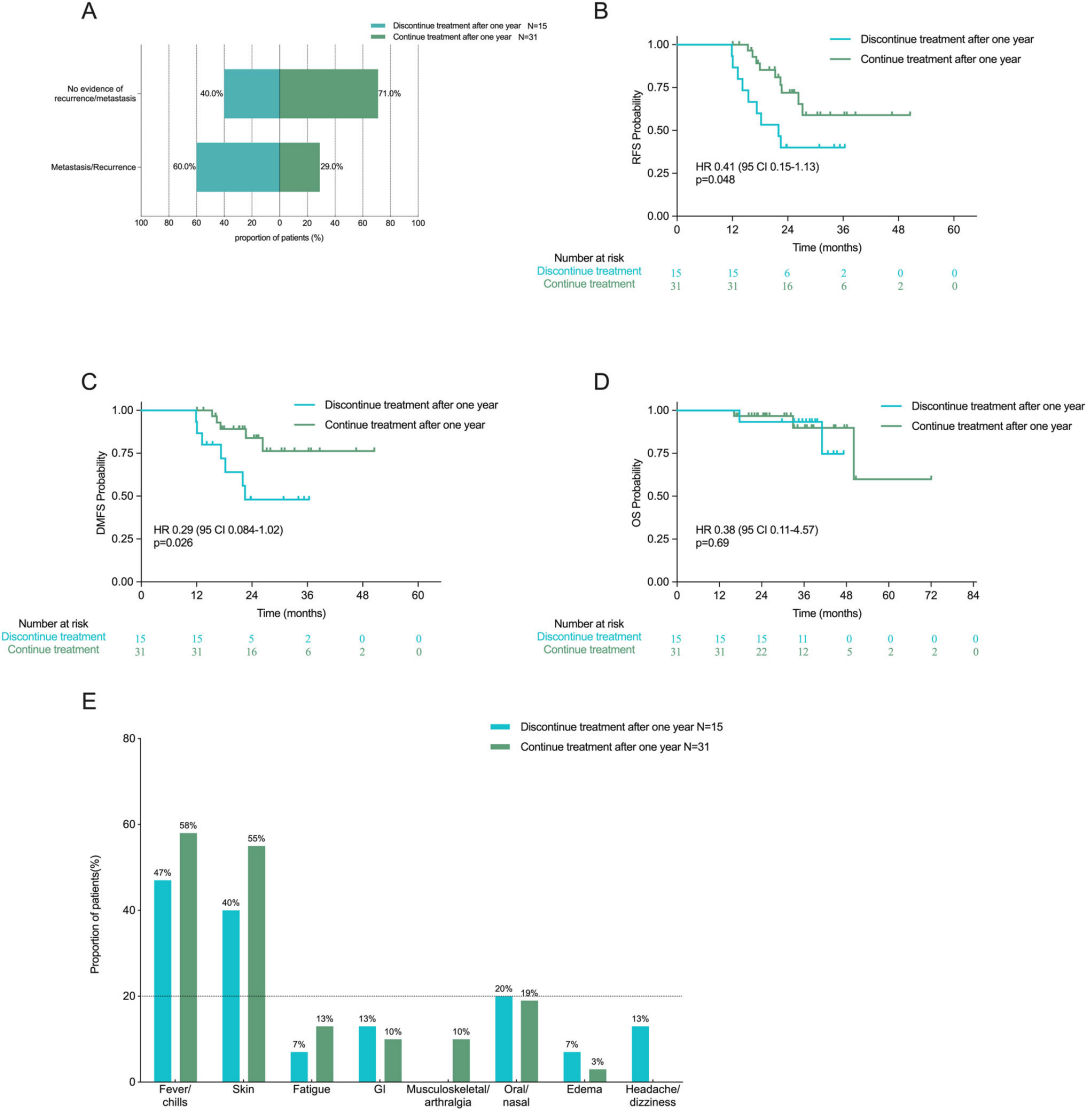
Separate analysis of patients who continued D/T therapy beyond 1 year versus those who followed the standard 1‐year D/T treatment duration. (A) The recurrence/metastasis ratio of the two groups at the time of the last follow‐up. (B) RFS between standard 1‐year treatment and continued treatment beyond 1 year (*p* = 0.048). (C) DMFS between standard 1‐year treatment and continued treatment beyond 1 year (*p* = 0.026). (D) OS between standard 1‐year treatment and continued treatment beyond 1 year (*p* = 0.69). (E) Detailed adverse events with proportions. DMFS, distant metastasis‐free survival; D/T, dabrafenib/trametinib; OS, overall survival; RFS, relapse‐free survival.

Among patients who discontinued therapy within 1 year, disease recurrence or metastasis was the predominant cause (Figure 3B). In the aPD‐1 cohort that completed the full 12‐month treatment (*n* = 7), the median RFS reached 28.1 months (95% CI 14.5‐NR), while the median DMFS was not reached (95% CI 15.5‐NR). Among patients who completed 1 year of D/T therapy, significant outcome disparities emerged based on treatment continuation. Baseline characteristics of the two subgroups were comparable, as summarized in Table S6. Those who continued treatment beyond the standard 1‐year duration (median treatment duration 20.0 months, 95% CI 17.5–23.4; a subset of patients remained on D/T therapy at the data cutoff) exhibited substantially lower rates of recurrence/metastasis compared to patients who discontinued treatment after 1 year treatment (median treatment duration 12.2 months, 95% CI 22.7‐NR; median time to progression post‐discontinuation 3.5 months, 95% CI 1.0‐NR) (Figure 4A). This disparity translated into clinically meaningful differences in RFS and DMFS (Figure 4B,C and Table 3). No significant difference in OS was observed (*p* = 0.69) (Figure 4D), with median OS unreached in both subgroups.

**TABLE 3:** RFS and DMFS comparison between patients who discontinued or continued D/T treatment after 1 year.

	Discontinue treatment after 1 year (*n* = 15)	Continue treatment after 1 year (*n* = 31)
**RFS**		
**Median (months, 95% CI)**	22.0 (15.5–NR)	NR (26.3–NR)
2‐year rate (%, 95% CI)	40.0 (21.5–74.3)	72.0 (56.2–92.2)
3‐year rate (%, 95% CI)	40.0 (21.5–74.3)	58.9 (40.6–85.5)
*p*‐value	0.048	
**DMFS**		
**Median (months, 95% CI)**	22.5 (18.2–NR)	NR (NR–NR)
2‐year rate (%, 95% CI)	48.0 (27.3–84.5)	83.9 (70.3–100.0)
3‐year rate (%, 95% CI)	48.0 (27.3–84.5)	76.3 (59.0–98.6)
*p*‐value	0.026	

Abbreviations: DMFS, distant metastasis‐free survival; NR, not reached; RFS, relapse‐free survival.

Targeted next‐generation sequencing (NGS) was conducted on tumor samples from a cohort of 24 patients, which included eight individuals from the interferon group, two from the aPD‐1 group, four from the D/T group, and 10 from the BRAFi/aPD‐1 group. The analysis concentrated on the correlation between gene mutations and patient survival outcomes, as illustrated in Figure S4. The findings indicated that more than half of the patients with secondary driver mutations in the *NOTCH4* gene experienced improved survival outcomes, with OS exceeding 3 years, irrespective of the adjuvant therapy administered. In the BRAFi/aPD‐1 group, most patients with concurrent mutations in the interleukin‐7 receptor (IL7R) gene also showed prolonged survival. Notably, all evaluable patients (*n* = 6) were classified as having a low tumor mutational burden (TMB‐L, defined as fewer than 10 mutations per megabase).

### 2.3 Adverse Events (AEs)

Grade ≥ 3 AEs were exclusively reported in the interferon group, affecting seven out of 25 patients (28%). These included myelosuppression (5/25 [20%]), hepatotoxicity (1/25 [4%]), and skin toxicity (1/25 [4%]). Persistent AEs were predominantly noted in the aPD‐1 group, with a significant impact on the endocrine system (Table S7). Although the BRAFi/aPD‐1 group exhibited the highest overall incidence of AEs, these were primarily transient and could be managed through dose modifications. The D/T group experienced a broader spectrum of AEs, particularly chills and fever, with some cases necessitating temporary treatment interruptions, as detailed in Table 4. A comparative analysis of patients who completed 1 year of D/T therapy versus those who discontinued after 1 year revealed similar rates of toxicities (Figure 4E). Comprehensive distributions of AEs across all treatment arms are provided in Figure S2.

**TABLE 4:** AEs with consequence in four cohorts.

AE type, *n* (%)	Interferon	aPD‐1	D/T	BRAFi/aPD‐1
	(*n* = 25)	(*n* = 18)	(*n* = 62)	(*n* = 17)
Any AE	14 (33)	10 (56)	47 (76)	16 (94)
Any dose interruptions/modifications due to AE	5 (20)	1 (6)	6 (10)	13 (76)
Discontinuation due to AE	4 (16)	1 (6)	2 (3)	1 (6)
Persistent AE	1 (4)	2 (11)	0 (0)	1 (6)

Abbreviations: AE, adverse event; aPD‐1, anti‐program death‐1 antibody; BRAFi, BRAF inhibitor; D/T, dabrafenib/trametinib.

## 3 Discussion

The results of this investigation suggest that adjuvant therapy with dabrafenib and trametinib (D/T) may represent the optimal treatment strategy for stage III Chinese melanoma patients harboring BRAF V600 mutations. Prolonging D/T treatment beyond 1 year appears to yield additional benefits. While RFS with aPD‐1 immunotherapy was inferior to those of D/T, the non‐inferior DMFS and OS outcomes may suggest specific clinical benefits of aPD‐1 blockade in patients with localized early‐stage melanoma. The biomarkers NOTCH4 and IR 7R are anticipated to serve as prognostic and predictive indicators for long‐term survival and the efficacy of targeted‐immunotherapy combinations, respectively.

In this cohort study, adjuvant interferon therapy did not demonstrate RFS benefits comparable to those observed in the historical EST 1684 trial [15], which may be attributed to the inherently elevated postoperative recurrence risk in patients with BRAF V600 mutations. In our Chinese cohorts, both aPD‐1 and D/T exhibited inferior RFS and DMFS compared to their counterparts in the KEYNOTE‐054 and COMBI‐AD trials [5, 16]. Notably, completion of 12 months of aPD‐1 therapy was associated with outcomes comparable to Western benchmarks, while for D/T, similar benefits were mainly seen in patients who continued treatment beyond the standard 12‐month course. In routine clinical practice, the 12‐month adjuvant regimen was consistently implemented according to current guidelines. During follow‐up discussions, patients were informed that postoperative relapse risk in resected stage III melanoma tends to cluster within the first 18–24 months after surgery [17, 18, 19]. Against this background, some recurrence‐free patients who completed the standard 12‐month D/T course with acceptable tolerability proactively requested treatment continuation because of persistent concern about relapse. Following detailed counseling regarding potential benefits, uncertainties, and cumulative toxicity, treatment extension was pursued only after an informed, patient‐led decision. Importantly, prolonging D/T beyond 12 months did not result in a significantly higher incidence of treatment‐related AEs compared with standard‐duration therapy. However, due to the limited data available, the optimal duration of D/T treatment remains undetermined, necessitating future large‐scale prospective cohort studies to address this question.

These findings suggest that the comparatively less favorable therapeutic responses observed in Chinese patients may be attributable to multiple clinical and biological factors—including differences in histologic subtype distribution, baseline tumor burden, LDH levels, and treatment adherence—emphasizing the necessity for personalized treatment strategies tailored to this population. It is noteworthy that the aPD‐1 cohort predominantly exhibited local recurrences while maintaining non‐inferior control of distant metastases compared to D/T, despite poorer RFS outcomes. Although stage‐stratified subgroup analyses were limited by the predominance of stage III C/unspecified patients across cohorts, our findings are generally consistent with the subgroup analyses from the KEYNOTE‐054 and COMBI‐AD studies, which recommend targeted therapy for patients in the locally advanced stage and suggest that aPD‐1 immunotherapy may offer unique advantages in controlling distant metastasis for patients in the localized early stage [5, 16].

While the combination of immunotherapy with BRAF/MEK inhibitors has not yet been established as a standard adjuvant treatment option, the solid efficacy of BRAF/MEK inhibitors in the early‐stage melanoma, coupled with the distinct advantages of aPD‐1 in long‐term DMFS, indicates that combination therapy may harness the benefits of both modalities [16, 20]. Preclinical models also suggest that BRAF and MEK inhibitors can enhance the anti‐tumor activity of immunotherapy [21, 22]. Consequently, a BRAFi/aPD‐1 combination regimen was strategically adopted as an exploratory adjuvant therapeutic approach. For Chinese patients who are unable to tolerate the febrile adverse reactions associated with D/T or who have contraindications to MEK inhibitors due to existing ocular or cardiac comorbidities, the combination of BRAF inhibitors and aPD‐1 may represent a viable alternative. Our findings indicate occurred effectively managed through dose modification and did not substantially compromise treatment adherence; nevertheless, clinicians should remain vigilant regarding short‐term toxicities and the potential for persistent immune‐related adverse effects with prolonged aPD‐1 exposure.

Genetic analyses indicate that secondary NOTCH4 driver mutations may serve as predictive biomarkers for long‐term survival in BRAF‐mutant melanoma. This association could be attributed to increased TMB and neoantigen load mediated by NOTCH4 alterations, as well as enhanced antigen presentation and co‐stimulatory pathway activation [23]. Additionally, prior evidence has shown that elevated IL7R expression marks an epigenetically primed memory CD8+ T‐cell population that is critical for antitumor immunity, and the IL7R signaling pathway is essential for sustaining responsiveness to immune checkpoint inhibitors [24, 25]. These findings, along with our analyses, may position IL7R alterations as potential combinatorial biomarkers for the integration of targeted therapy and immunotherapy. It is important to note that these results were obtained from a limited NGS subset, selected mainly based on tissue availability, which led to a small sample size and an imbalanced representation among treatment groups. Therefore, the associations involving NOTCH4 and IL7R should be considered preliminary and interpreted with caution. Prospective clinical trials are warranted to validate their clinical utility.

Given the retrospective and non‐randomized nature of this study, as well as the limited sample size, the conclusions drawn should be interpreted with caution. In particular, the relatively small sample sizes of the aPD‐1 and BRAFi/aPD‐1 cohorts restrict the robustness of the efficacy assessments in these subgroups; consequently, the related findings should therefore be interpreted with caution. Furthermore, with a median follow‐up duration of 21 months, this study has limited capacity to provide insights into long‐term outcomes for these patients. Accordingly, the absence of statistically significant differences in OS should be regarded as preliminary, and longer follow‐up will be necessary to determine whether true OS divergence emerges among the treatment groups. Despite these limitations, multivariate analyses indicate that adjuvant D/T therapy and the combination of BRAF inhibitors with aPD‐1 therapy were independent favorable predictors across both Cox proportional hazards and RMST models. Additionally, elevated baseline SAA levels appeared to be a stronger predictor of increased DMFS risk than RFS risk, suggesting that SAA may serve as a potential prognostic biomarker in future clinical practice.

Due to the fundamentally different mechanisms of these therapies, longer follow‐up durations will be necessary to validate these findings. Ideally, large‐scale, prospective, randomized controlled trials should be conducted to confirm our results and establish optimized treatment strategies.

## 4 Conclusions

For Chinese patients with stage III melanoma harboring BRAF‐V600 mutations, adjuvant D/T therapy may provide the most significant clinical benefit. Furthermore, to maximize therapeutic efficacy in this population, extending D/T therapy beyond 1 year may be advisable. Interferon adjuvant therapy demonstrated limited benefits. For specific patient groups in China, the combination of targeted therapy and immunotherapy is also a viable option, offering survival benefits comparable to those of D/T, with good tolerance for related adverse reactions. Secondary NOTCH4 and IL7R driver mutations may respectively serve as potential biomarkers for the prognosis and treatment selection of melanoma patients.

## 5 Materials and Methods

### 5.1 Patients and Treatment

This investigation encompassed patients diagnosed with stage III melanoma, aged 18 years and older, who exhibited BRAF V600 mutations and had undergone surgical resection of either primary or metastatic lesions. From June 2013 to December 2023, these individuals received adjuvant therapy post‐surgery at three melanoma treatment centers in China: Sun Yat‐sen University Cancer Center, Yunnan Cancer Hospital, and Huazhong University of Science and Technology Union Shenzhen Hospital. Participants were categorized into four distinct cohorts based on the type of adjuvant therapy administered: (1) high‐dose interferon, (2) monotherapy with anti‐PD‐1 antibody (aPD‐1), (3) dabrafenib plus trametinib (D/T), and (4) a regimen combining a BRAF inhibitor with aPD‐1 (BRAFi/aPD‐1). In the BRAFi/aPD‐1 group, a planned targeted lead‐in with BRAFi monotherapy was implemented to improve tolerability and reduce early toxicity. For this group, the duration of adjuvant therapy was calculated from the initiation of BRAFi treatment. Vemurafenib was predominantly used as the BRAF inhibitor because it was the first agent widely available in China during the early study period, prior to the introduction of dabrafenib.

Data collection was conducted independently at each center, followed by organization and processing for subsequent analysis. Patient demographics, disease characteristics, as well as baseline SAA levels were gathered and analyzed. Baseline SAA levels were interpreted in accordance with the institutional reference range of 0–10 mg/L. The primary endpoints were RFS and DMFS. RFS was operationally defined as the interval from the final surgical intervention to the occurrence of local recurrence, distant metastasis, death, or the last follow‐up, whichever transpired first. Conversely, DMFS was defined as the duration from definitive surgery to the emergence of any distant metastasis or death, whichever occurred first. Secondary endpoints included OS, which was defined as the time from the final surgical procedure to death or the last follow‐up, whichever occurred first. Recurrence and metastasis were determined based on radiologic assessment using RECIST (Response Evaluation Criteria In Solid Tumors) v1.1 criteria and the clinical judgment of the attending physician. Local recurrence encompassed relapse at the primary tumor or surgical scar, in‐transit metastasis, and regional lymph node recurrence. AEs were graded by the treating physician at the time of occurrence and subsequently reviewed independently by medical oncologists to ensure quality control and appropriate attribution to the study treatment. The severity of treatment‐related AEs was assessed using the National Cancer Institute Common Terminology Criteria for Adverse Events (CTCAE) version 4.0, consistent with the documentation standards used across participating centers during the study period. In accordance with the consensus definition for immune‐related adverse events (irAEs) established by the *Journal for Immunotherapy of Cancer* (*JITC*) [26], persistent irAEs were defined as those that remained present for more than 3 months following the cessation of treatment. The same criteria were applied to AEs associated with D/T [27].

### 5.2 Targeted Next‐Generation Sequencing

Formalin‐fixed paraffin‐embedded (FFPE) tumor tissue specimens—including primary, recurrent, and metastatic lesions—were obtained from patients treated at Sun Yat‐sen University Cancer Center (SYSUCC) and were collected and sectioned into 4–5 µm slices. Selection of tumor samples for NGS was primarily based on tissue availability and sample quality, rather than on recurrence status or treatment response, to ensure sufficient DNA yield for sequencing. Genomic DNA was extracted utilizing the DNA FFPE Tissue Kit (Tiangen, China). Targeted NGS was conducted in the clinical laboratory of the Department of Molecular Diagnostics at SYSUCC employing a pan‐cancer 1021‐gene panel (GenePlus Institute, Beijing, China) that encompasses 1021 cancer‐associated genes [28]. Sequencing was performed on the GenePlus 2000 system (GenePlus Institute, China) utilizing 100‐bp paired‐end reads. The clinical significance of somatic sequence variants was classified in accordance with the Standards and Guidelines for the Interpretation and Reporting of Sequence Variants in Cancer [29].

### 5.3 Statistical Analysis

Categorical variables were summarized as frequencies and percentages, while continuous variables were expressed as medians with IQR. Baseline categorical characteristics across the four treatment groups were compared using the chi‐square test (or the Fisher–Freeman–Halton exact test when the expected cell count was less than five). For continuous variables, comparisons were performed using the Kruskal–Wallis *H* test. All primary and secondary endpoints were evaluated. Survival outcomes, including RFS, DMFS, and OS, were analyzed through Kaplan–Meier survival curves.

Univariate comparisons were executed using the log‐rank test and Cox proportional hazards regression models. Variables exhibiting a moderate association with the outcome (defined as *p* < 0.2; for categorical variables with more than two categories, at least one category with *p* < 0.2) were incorporated into the multivariate Cox regression analysis [27]. In instances where the proportional hazards assumption was not satisfied, RMST was utilized as a non‐parametric measure. RMST is defined as the area under the survival curves prior to a predetermined time point [30]. Considering the temporal distribution of recurrence events in this cohort and a median follow‐up duration of 21 months, a 2‐year truncation point was chosen. This interval encompassed the majority of RFS and DMFS events, thereby offering a clinically meaningful and statistically robust timeframe for RMST comparison. Accordingly, a pre‐specified secondary RMST analysis was performed at the 2‐year landmark for RFS, DMFS, and OS [31]. All statistical tests were two‐sided (or double one‐sided for Fisher's exact test), with a significance threshold set at *p* < 0.05. All analyses were performed using R version 4.2.2 and GraphPad Prism version 10.3.1.

## Author Contributions

The study conception and design were collaboratively developed by Xiaoshi Zhang and Dandan Li. Administrative support was provided by Baiwei Zhao, Wei Zheng, Ke Li, and Qiming Zhou. Data collection was conducted by Rongcheng Zhang, Qiong Zhang, Ziluan Chen, Renai Li, and Qianqi Chen. Data analysis and interpretation were performed by Rongcheng Zhang, Yao Liang, Jingjing Li, Junwan Wu, Qiong Zhang, Qiuyue Ding, and Linbin Chen. Dandan Li, Xiaoshi Zhang, Ke Li, Qiming Zhou, Xizhi Wen, and Ya Ding accessed and validated the data. The manuscript was written by Rongcheng Zhang, Yao Liang, Jingjing Li, and Qianqi Chen. Dandan Li was responsible for the overall content. All authors have read and approved the final manuscript.

## Funding

This study was funded by the National Natural Science Foundation of China (Grant No. 82202179), Guangdong Basic and Applied Basic Research Foundation (Grant No. 2024A1515010391), Guangdong Basic and Applied Basic Research Foundation (Grant No. 2021A1515010099), Young Talents Program of Sun Yat‐sen University Cancer Center (Grant No. YTP‐SYSUCC‐0026), Beijing Xisike Clinical Oncology Research Foundation (Grant No. Y‐JS2019‐101), and Wu Jieping Medical Foundation (Grant No. 320.6750.2024‐16‐6). The funders played no role in any aspect of the study, including the design, data collection, analysis, or interpretation of data, or the writing of this manuscript.

## Ethics Statement

This study was conducted with the approval of the Ethics Committee of Sun Yat‐sen University Cancer Center (B2025‐319) and performed in accordance with the Declaration of Helsinki. A waiver for informed consent was obtained.

## Conflicts of Interest

The authors declare no conflicts of interest.

## Supporting information




**Figure S1**. OS of adjuvant Interferon, aPD‐1, D/T and BRAFi/aPD‐1 treated patients with resected BRAF V600 mutant melanoma. A) OS between D/T and Interferon (p = 0.22). B) RFS between D/T and aPD‐1 (p = 0.93). C) RFS between D/T and BRAFi/aPD‐1 (p = 0.85). D) RFS between aPD‐1 and BRAFi/aPD‐1 (p = 0.58). D/T, dabrafenib/trametinib; aPD‐1, anti‐program death‐1 antibody; BRAFi, BRAF inhibitor; RFS, relapse free survival; OS, overall survival.
**Figure S2**. Detailed adverse events and their proportions in patients with BRAF V600‐mutated melanoma resected with adjuvant interferon, aPD‐1, D/T, and BRAFi/aPD‐1 therapy. D/T, dabrafenib/trametinib; aPD‐1, anti‐program death‐1 antibody; BRAFi, BRAF inhibitor.
**Figure S3**. The distribution of metastatic sites across treatment groups. D/T, dabrafenib/trametinib; aPD‐1, anti‐program death‐1 antibody; BRAFi, BRAF inhibitor.
**Figure S4**. Distribution and survival association of recurrently mutated genes across different treatment groups. The heatmap shows somatic gene mutations identified by targeted next‐generation sequencing (NGS) that were present in at least two of the four treatment groups: Interferon, anti–PD‐1 (aPD‐1), D/T (dabrafenib + trametinib), and BRAFi/aPD‐1 (BRAF inhibitor combined with anti–PD‐1 therapy). Each cell indicates the presence and frequency of a given mutation within a treatment group, with color intensity proportional to the number of patients harboring that mutation. Symbols within cells represent the survival status associated with the mutation: “+” indicates that more than half of the patients carrying the mutation achieved long overall survival (OS > 3 years); “−” indicates that fewer than half had long survival; and “+/−” indicates an equal distribution between long and short survival.
**Table S1**. OS by different adjuvant therapies.
**Table S2**. Univariate and multivariate analyses of RFS (Cox model).
**Table S3**. Univariate and multivariate analyses of DMFS (Cox model).
**Table S4**. Univariate and multivariate analyses of OS (Cox model).
**Table S5**. 2‐year RMST by different adjuvant therapies.
**Table S6**. Baseline characteristics of patients who completed the standard 1‐year D/T regimen.
**Table S7**. Most common AEs (incidence) that led to discontinuation, treatment modification, or persisted.

## Data Availability

The datasets used or analyzed in the current study are available from the corresponding authors upon reasonable request. Raw next‐generation sequencing data have been deposited in Genome Sequence Archive of National Genomics Data Center with open access (bioProject accession:PRJCA039909), according to Guidance of the Ministry of Science and Technology (MOST) for the Review and Approval of Human Genetic Resources (https://bigd.big.ac.cn/gsa‐human/browse/HRA013175).
