# Supplementary material for: Optimal Adjuvant Therapy Selection for Chinese BRAF V600‐Mutant Stage III Melanoma: A Multicenter Efficacy Comparison of Targeted Agents, Immunotherapy, and Combinatorial Strategies
Source: MedComm (2020). 2026 Apr 25;7(5):e70738. doi: 10.1002/mco2.70738 (PMC13109861; doi:10.1002/mco2.70738)
Supplement: Supplementary file 1 — Figure S1. OS of adjuvant Interferon, aPD‐1, D/T and BRAFi/aPD‐1 treated patients with resected BRAF V600 mutant melanoma. A) OS between D/T and Interferon (p = 0.22). B) RFS between D/T and aPD‐1 (p = 0.93). C) RFS between D/T and BRAFi/aPD‐1 (p = 0.85). D) RFS between aPD‐1 and BRAFi/aPD‐1 (p = 0.58). D/T, dabrafenib/trametinib; aPD‐1, anti‐program death‐1 antibody; BRAFi, BRAF inhibitor; RFS, relapse free survival; OS, overall survival. Figure S2. Detailed adverse events and their proportions in patients with BRAF V600‐mutated melanoma resected with adjuvant interferon, aPD‐1, D/T, and BRAFi/aPD‐1 therapy. D/T, dabrafenib/trametinib; aPD‐1, anti‐program death‐1 antibody; BRAFi, BRAF inhibitor. Figure S3. The distribution of metastatic sites across treatment groups. D/T, dabrafenib/trametinib; aPD‐1, anti‐program death‐1 antibody; BRAFi, BRAF inhibitor. Figure S4. Distribution and survival association of recurrently mutated genes across different treatment groups. The heatmap shows somatic gene mutations identified by targeted next‐generation sequencing (NGS) that were present in at least two of the four treatment groups: Interferon, anti–PD‐1 (aPD‐1), D/T (dabrafenib + trametinib), and BRAFi/aPD‐1 (BRAF inhibitor combined with anti–PD‐1 therapy). Each cell indicates the presence and frequency of a given mutation within a treatment group, with color intensity proportional to the number of patients harboring that mutation. Symbols within cells represent the survival status associated with the mutation: “+” indicates that more than half of the patients carrying the mutation achieved long overall survival (OS > 3 years); “−” indicates that fewer than half had long survival; and “+/−” indicates an equal distribution between long and short survival. Table S1. OS by different adjuvant therapies. Table S2. Univariate and multivariate analyses of RFS (Cox model). Table S3. Univariate and multivariate analyses of DMFS (Cox model). Table S4. Univariate and multivariate anal [file MCO2-7-e70738-s001.docx]

**Supplementary Materials**

**Optimal Adjuvant Therapy Selection for Chinese BRAF V600-Mutant Stage III Melanoma: A Multicenter Efficacy Comparison of Targeted Agents, Immunotherapy, and Combinatorial Strategies**

Rongcheng Zhang ^1, #^, Yao Liang ^2, 5, #^, Jingjing Li ^1, #^, Qianqi Chen ^3, #^, Ya Ding ^1^, Xizhi Wen ^1^, Baiwei Zhao ^4, 5^, Wei Zheng ^6^, Junwan Wu ^1^, Qiong Zhang ^1^, Ziluan Chen ^1^, Qiuyue Ding ^1^, Linbin Chen ^1^, Renai Li ^1^, Ke Li ^7, *^, Qiming Zhou ^3, *^, Xiaoshi Zhang ^1, *^, Dandan Li ^1, *^

**Author details**

^1^ Biotherapy Center, Sun Yat-sen University Cancer Center, State Key Laboratory of Oncology in South China, Guangdong Provincial Clinical Research Center for Cancer, Collaborative Innovation Center for Cancer Medicine, Guangzhou, Guangdong Province 510060, P. R. China

^2^ Sun Yat-Sen University Cancer Center, State Key Laboratory of Oncology in South China, Collaborative Innovation Center for Cancer Medicine, Guangdong Province 510060, P. R. China

^3^ Department of Oncology, Huazhong University of Science and Technology Union Shenzhen Hospital, Shenzhen, Guangdong Province 518052, P. R. China

^4^ State Key Laboratory of Oncology in South China, Guangdong Provincial Clinical Research Center for Cancer，Sun Yat-sen University Cancer Center, Guangdong Province 510060, P. R. China

^5^ Department of Gastric Surgery, Sun Yat-sen University Cancer Center, Guangzhou 510060, P. R. China

^6^ Department of Ultrasound, Sun Yat-Sen University Cancer Center, State Key

^7^ Department of Cancer Biotherapy Center, Yunnan Cancer Hospital, The Third Affiliated Hospital of Kunming Medical University, Cancer Center of Yunnan Province, Kunming, Yunnan Province 650118, P. R. China

^#^ Rongcheng Zhang, Yao Liang, Jingjing Li and Qianqi Chen contributed equally to this work

^*^ Corresponding authors

**Correspondence:**

Dandan Li and Xiaoshi Zhang, Biotherapy Center, Sun Yat-sen University Cancer Center, Guangzhou, Guangdong Province, P. R. China. E-mail address: [lidd@sysucc.org.cn](mailto:lidd@sysucc.org.cn) and [zhangxsh@sysucc.org.cn](mailto:zhangxsh@sysucc.org.cn)

Qiming Zhou: Department of Oncology, Huazhong University of Science and Technology Union Shenzhen Hospital, Shenzhen, Guangdong Province, P. R. China. E-mail address: [zqm961221@163.com](mailto:zqm961221@163.com)

Ke Li: Department of Cancer Biotherapy Center, Yunnan Cancer Hospital, Kunming, Yunnan Province, P. R. China. E-mail address: [likelikelike@126.com](mailto:likelikelike@126.com)

**Supplementary Materials**

**Figure S1.** OS of adjuvant Interferon, aPD-1, D/T and BRAFi/aPD-1 treated patients with resected BRAF V600 mutant melanoma.

**Figure S2.** Detailed adverse events and their proportions in patients with BRAF V600-mutated melanoma resected with adjuvant interferon, aPD-1, D/T, and BRAFi/aPD-1 therapy.

**Figure S3.** The distribution of metastatic sites across treatment groups.

**Figure S4.** Distribution and survival association of recurrently mutated genes across different treatment groups.

**Table S1.** OS by different adjuvant therapies.

**Table S2.** Univariate and multivariate analyses of RFS (Cox model).

**Table S3.** Univariate and multivariate analyses of DMFS (Cox model).

**Table S4.** Univariate and multivariate analyses of OS (Cox model).

**Table S5.** 2-year RMST by different adjuvant therapies.

**Table S6.** Baseline characteristics of patients who completed the standard 1-year D/T regimen.

**Table S7.** Most common AEs (incidence) that led to discontinuation, treatment

modification, or persisted.


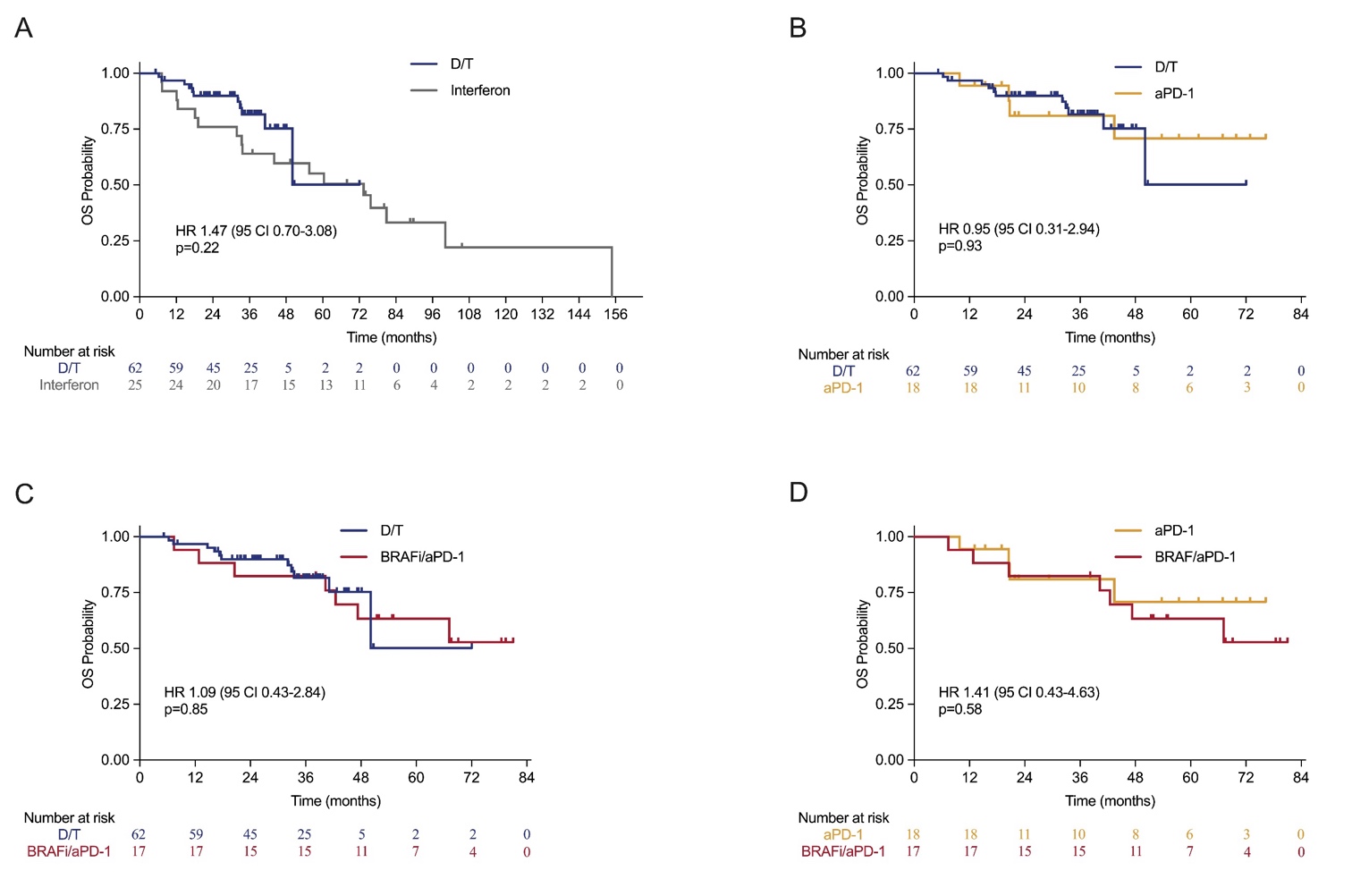


**Figure S1.** OS of adjuvant Interferon, aPD-1, D/T and BRAFi/aPD-1 treated patients with resected BRAF V600 mutant melanoma. A) OS between D/T and Interferon (P = 0.22). B) RFS between D/T and aPD-1 (P = 0.93). C) RFS between D/T and BRAFi/aPD-1 (P = 0.85). D) RFS between aPD-1 and BRAFi/aPD-1 (P = 0.58). D/T, dabrafenib/trametinib; aPD-1, anti-program death-1 antibody; BRAFi, BRAF inhibitor; RFS, relapse free survival; OS, overall survival.


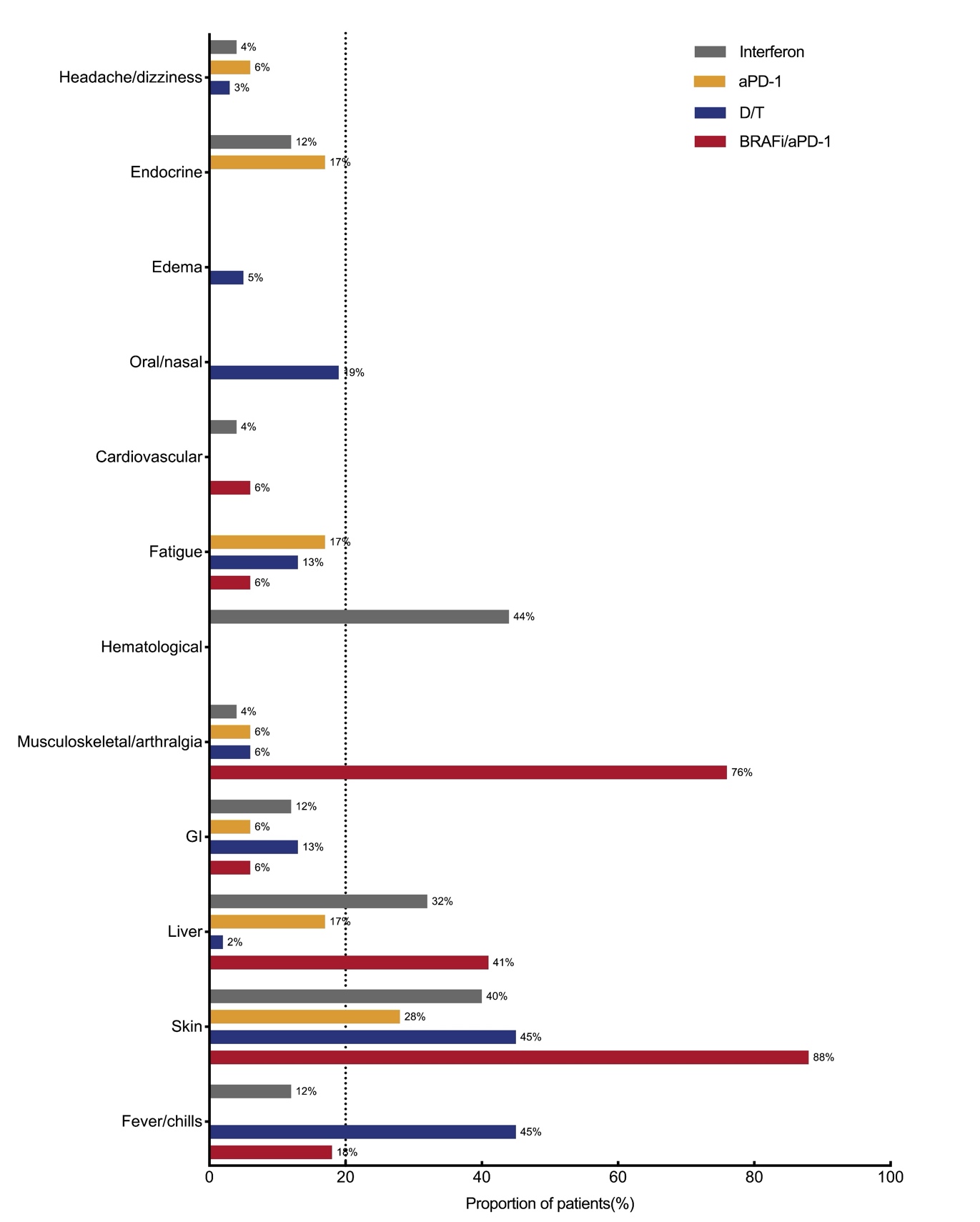


**Figure S2.** Detailed adverse events and their proportions in patients with BRAF V600-mutated melanoma resected with adjuvant interferon, aPD-1, D/T, and BRAFi/aPD-1 therapy. D/T, dabrafenib/trametinib; aPD-1, anti-program death-1 antibody; BRAFi, BRAF inhibitor.


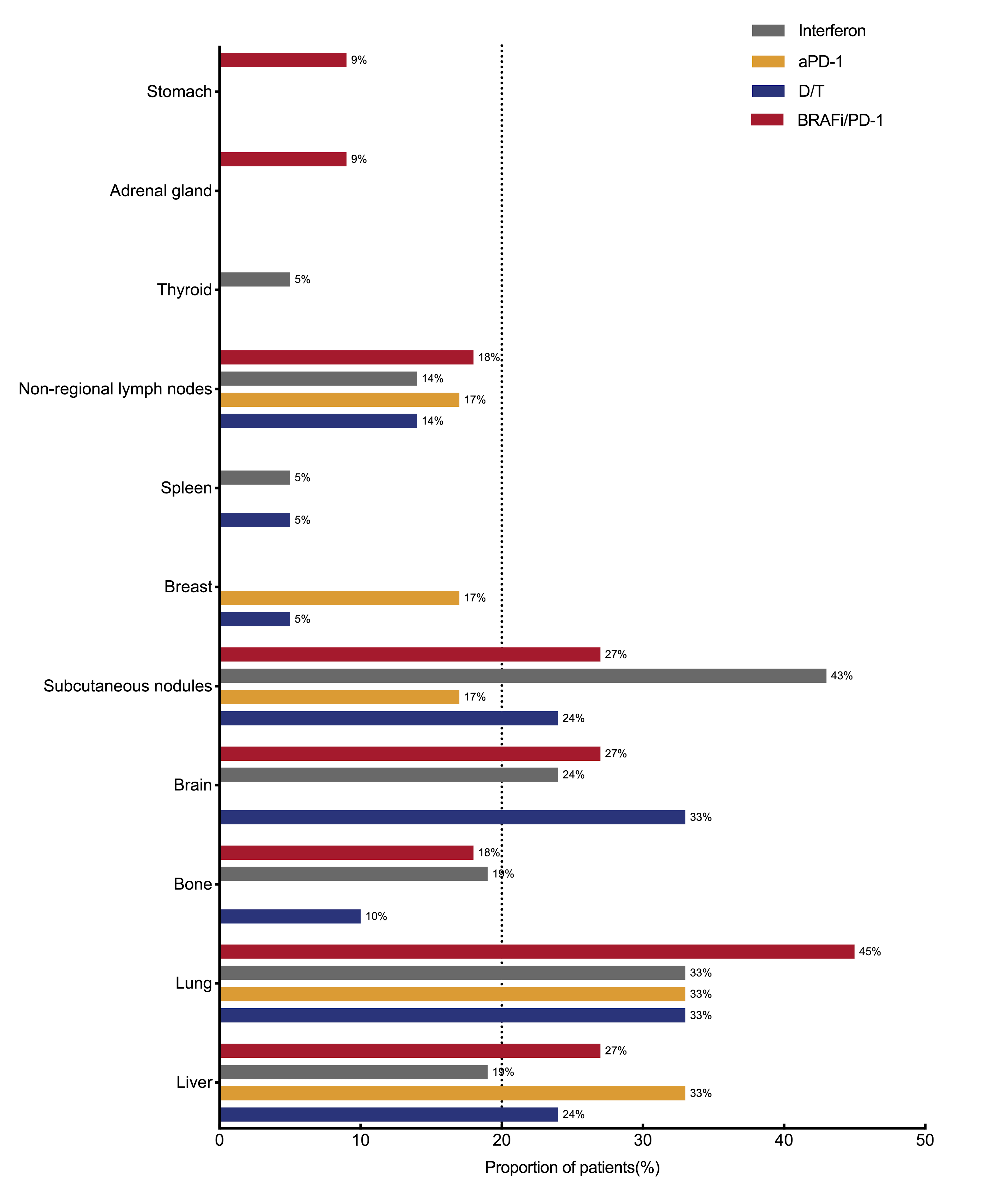


**Figure S3.** The distribution of metastatic sites across treatment groups. D/T, dabrafenib/trametinib; aPD-1, anti-program death-1 antibody; BRAFi, BRAF inhibitor.


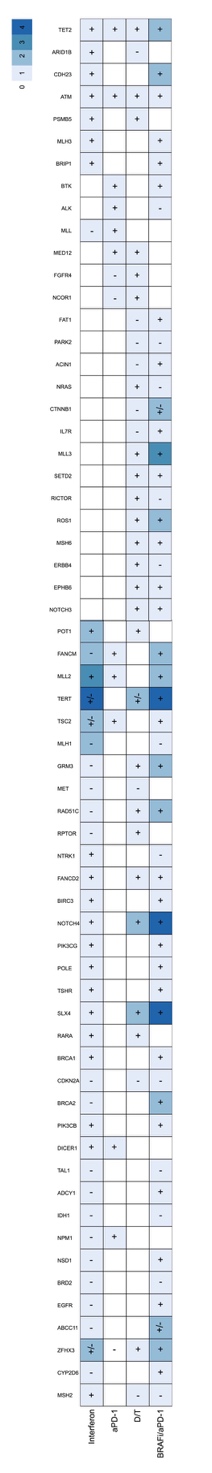


**Figure S4.** Distribution and survival association of recurrently mutated genes across different treatment groups. The heatmap shows somatic gene mutations identified by targeted next-generation sequencing (NGS) that were present in at least two of the four treatment groups: Interferon, anti–PD-1 (aPD-1), D/T (dabrafenib + trametinib), and BRAFi/aPD-1 (BRAF inhibitor combined with anti–PD-1 therapy). Each cell indicates the presence and frequency of a given mutation within a treatment group, with color intensity proportional to the number of patients harboring that mutation. Symbols within cells represent the survival status associated with the mutation: “+” indicates that more than half of the patients carrying the mutation achieved long overall survival (OS > 3 years); “−” indicates that fewer than half had long survival; and “+/−” indicates an equal distribution between long and short survival.

**Table S1.** OS by different adjuvant therapies.

|  | **Interferon** | **aPD-1** | **D/T** | **BRAFi/aPD-1** |
| --- | --- | --- | --- | --- |
|  | **(n=25)** | **(n=18)** | **(n=62)** | **(n=17)** |
| **OS** |  |  |  |  |
| **Median (months, 95%CI)** | **73.4 (33.7-NR)** | **NR (43.4-NR)** | **NR (40.1-NR)** | **NR (67**.2**-NR)** |
| 1-y rate (%, 95%CI) | 92.0 (82.0-100.0) | 94.4 (84.4-100.0) | 96.7 (92.4-100.0) | 93.8 (82.6-100.0) |
| 3-y rate (%, 95%CI) | 64.0 (47.7-85.9) | 81.0 (63.6-100.0) | 81.6 (71.0-93.8) | 81.3 (64.2-100.0) |
| 5-y rate (%, 95%CI) | 55.1 (38.5-79.0) | 70.8 (49.6-100.0) | 52.6 (28.6-96.6) | 50.2 (22.0-100.0) |
| **P-value** | **0.22** | **0.93** |  | **0.85** |

D/T, dabrafenib/trametinib; aPD-1, anti-program death-1 antibody; BRAFi, BRAF inhibitor; NR, not reached; OS, overall survival.

**Table S2.** Univariate and multivariate analyses of RFS (Cox model).

|  | **HR (95% CI)** | **P value** | **HR (95% CI)** | **P value** |
| --- | --- | --- | --- | --- |
| **Age** |  | 0.83 |  |  |
| <60 | 1 |  |  |  |
| ≥60 | 0.94 (0.53-1.67) |  |  |  |
| **Sex** |  | 0.52 |  |  |
| Male | 1 |  |  |  |
| Female | 0.87 (0.56-1.34) |  |  |  |
| **Melanoma subtype** |  | 0.14 |  | 0.2 |
| Non-acral cutaneous/Unknow primary | 1 |  | 1 |  |
| Non-cutaneous | 1.48 (0.88-2.50) |  | 1.42 (0.83-2.43) |  |
| **BRAF V600 mutation** |  |  |  |  |
| V600E | 1 |  |  |  |
| V600K | 1.01 (0.31-3.25) | 0.99 |  |  |
| V600E/K | 1.67 (1.04-2.65) | 0.032 |  |  |
| **SAA Level** |  |  |  |  |
| Elevated | 1.31 (0.75-2.32) | 0.36 |  |  |
| Normal | 1 |  |  |  |
| Unknow | 1.37 (0.83-2.26) | 0.22 |  |  |
| **Adjuvant therapy** |  |  |  |  |
| Interferon | 2.11 (1.21-3.68) | 0.008 | 1.99 (1.13-3.51) | 0.017 |
| aPD-1 | 2.56 (1.39-4.72) | 0.003 | 2.59 (1.41-4.79) | 0.0023 |
| D/T | 1 |  | 1 |  |
| BRAFi/aPD-1 | 1.26 (0.66-2.40) | 0.48 | 1.22 (0.64-2.33) | 0.55 |

D/T, dabrafenib/trametinib; aPD-1, anti-program death-1 antibody; BRAFi, BRAF inhibitor; RFS, relapse free survival.

**Table S3.** Univariate and multivariate analyses of DMFS (Cox model).

|  | **HR (95% CI)** | **P value** | **HR (95% CI)** | **P value** |
| --- | --- | --- | --- | --- |
| **Age** |  | 0.43 |  |  |
| <60 | 1 |  |  |  |
| ≥60 | 0.74 (0.35-1.57) |  |  |  |
| **Sex** |  | 0.4 |  |  |
| Male | 1 |  |  |  |
| Female | 0.80 (0.48-1.34) |  |  |  |
| **Melanoma subtype** |  | 0.17 |  | 0.17 |
| Non-acral cutaneous/Unknow primary | 1 |  | 1 |  |
| Non-cutaneous | 1.54 (0.83-2.87) |  | 1.72 (0.77-3.86) |  |
| **BRAF V600 mutation** |  |  |  |  |
| V600E | 1 |  |  |  |
| V600K | 0.53 (0.072-3.88) | 0.53 |  |  |
| V600E/K | 2.07 (1.21-3.54) | 0.008 |  |  |
| **SAA Level** |  |  |  |  |
| Elevated | 1.58 (0.83-2.99) | 0.16 | 1.83 (0.92-3.63) | 0.19 |
| Normal | 1 |  | 1 |  |
| Unknow | 1.09 (0.59-2.03) | 0.78 |  |  |
| **Adjuvant therapy** |  |  |  |  |
| Interferon | 2.73 (1.46-5.11) | 0.002 | 5.97 (2.50-14.27) | <0.001 |
| aPD-1 | 1.42 (0.57-3.52) | 0.46 | 2.20 (0.75-6.45) | 0.15 |
| D/T | 1 |  | 1 |  |
| BRAFi/aPD-1 | 1.49 (0.71-3.12) | 0.29 | 1.61 (0.67-3.86) | 0.28 |

D/T, dabrafenib/trametinib; aPD-1, anti-program death-1 antibody; BRAFi, BRAF inhibitor; DMFS, distant metastasis-free survival.

**Table S4.** Univariate and multivariate analyses of OS (Cox model).

|  | **HR (95% CI)** | **P value** | **HR (95% CI)** | **P value** |
| --- | --- | --- | --- | --- |
| **Age** |  | 0.8 |  |  |
| <60 | 1 |  |  |  |
| ≥60 | 0.90 (0.40-2.05) |  |  |  |
| **Sex** |  | 0.87 |  |  |
| Male | 1 |  |  |  |
| Female | 1.06 (0.55-2.03) |  |  |  |
| **Melanoma subtype** |  | 0.83 |  |  |
| Non-acral cutaneous/Unknow primary | 1 |  |  |  |
| Non-cutaneous | 1.09(0.48-2.50) |  |  |  |
| **BRAF V600 mutation** |  |  |  |  |
| V600E | 1 |  |  |  |
| V600K | 0.76 (0.10-5.64) | 0.79 |  |  |
| V600E/K | 1.41 (0.71-2.79) | 0.33 |  |  |
| **SAA Level** |  |  |  |  |
| Elevated | 0.70 (0.29-1.70) | 0.43 |  |  |
| Normal | 1 |  |  |  |
| Unknow | 0.85 (0.41-1.74) | 0.66 |  |  |
| **Adjuvant therapy** |  |  |  |  |
| Interferon | 1.80 (0.78-4.16) | 0.17 | 1.80 (0.78-4.16) | 0.17 |
| aPD-1 | 0.95 (0.30-3.02) | 0.93 | 0.95 (0.30-3.02) | 0.93 |
| D/T | 1 |  | 1 |  |
| BRAFi/aPD-1 | 1.30 (0.49-3.46) | 0.6 | 1.30 (0.49-3.46) | 0.6 |

D/T, dabrafenib/trametinib; aPD-1, anti-program death-1 antibody; BRAFi, BRAF inhibitor; OS, overall survival.

**Table S5.** 2-year RMST by different adjuvant therapies.

|  | **Interferon** | **aPD-1** | **D/T** | **BRAFi/aPD-1** |
| --- | --- | --- | --- | --- |
|  | **(n=25)** | **(n=18)** | **(n=62)** | **(n=17)** |
| **RFS** |  |  |  |  |
| **2-year RMST**  **(months, 95%CI)** | 13.4  (10.1-16.7) | 12.8  (9.1-16.5) | 19  (17.4-20.7) | 19.2  (16.1-22.3) |
| **P-value** | **0.02** | **0.01** |  | **0.9** |
| **DMFS** |  |  |  |  |
| **2-year RMST**  **(months, 95%CI)** | 13.9  (10.5-17.3) | 17.5  (13.5-21.5) | 20.2  (18.5-21.8) | 20.3  (17.3-23.4) |
| **P-value** | **<0.01** | **0.2** |  | **0.9** |
| **OS** |  |  |  |  |
| **2-year RMST**  **(months, 95%CI)** | 21.3  (19.2-23.4) | 22.8  (21.2-24.3) | 22.9  (22.0-23.8) | 22.2  (20.0-24.3) |
| **P-value** | **0.2** | **0.9** |  | **0.5** |

D/T, dabrafenib/trametinib; aPD-1, anti-program death-1 antibody; BRAFi, BRAF inhibitor; RMST, restricted mean survival time.

**Table S6.** Baseline characteristics of patients who completed the standard 1-year D/T regimen.

| **Characteristic** | **Discontinue**  **treatment**  **after 1 year  N = 15** | **Continue**  **treatment**  **after 1 year  N = 31** | **P-value** |
| --- | --- | --- | --- |
| **Age, Mean ± SD** | 47 ± 16 | 46 ± 14 | 0.875^1^ |
| **Sex, n (%)** |  |  | 0.886^2^ |
| Male | 5 (33.3) | 11 (35.5) |  |
| Female | 10 (66.7) | 20 (64.5) |  |
| **Melanoma subtype, n (%)** |  |  | 0.352^3^ |
| Non-acral cutaneous | 12 (80.0) | 26 (83.9) |  |
| Acral | 0 (0) | 3 (9.7) |  |
| Mucosal | 1 (6.7) | 1 (3.2) |  |
| Unknown primary | 2 (13.3) | 1 (3.2) |  |
| **Stage (AJCC 8th) of patients with cutaneous and acral melanoma, n (%)** |  |  | 0.231^3^ |
| IIIB | 1 (6.7) | 6 (19.4) |  |
| IIIC | 9 (60.0) | 10 (32.3) |  |
| IIID | 0 (0) | 4 (12.9) |  |
| III unspecified | 5 (33.3) | 11 (35.5) |  |
| **BRAF V600E/K mutation, n (%)** |  |  | 0.244^3^ |
| V600E | 13 (86.7) | 30 (96.8) |  |
| V600K | 1 (6.7) | 0 (0) |  |
| V600E/K | 1 (6.7) | 1 (3.2) |  |
| **SAA Level, n (%)** |  |  | >0.999^3^ |
| Normal | 5 (33.3) | 10 (32.3) |  |
| Elevated | 5 (33.3) | 11 (35.5) |  |
| Unknown | 5 (33.3) | 10 (32.3) |  |
| ^1^Welch Two Sample t-test,^2^Pearson's Chi-squared test,^3^Fisher's exact test. | | | |

**Table S7.** Most common AEs (incidence) that led to discontinuation, treatment modification, or persisted.

| **Group** | **AE type (number of patients, %)** | | |  |
| --- | --- | --- | --- | --- |
|  | **Permanent discontinuation** | **Treatment modification (both dose reduction and/or schedule interruption)** | **Persistent AE(s)** |  |
| **Interferon**  **n=25** | Skin (1,4%) | Skin (3,12%) | Skin (1,4%) |  |
|  | Liver (1,4%) | Cardiovascular (1,4%) |  |  |
|  | Others (1,4%) |  |  |  |
| **aPD-1**  **n=18** | Endocrine (1,6%) | Liver (1,6%) | Endocrine (2,11%) |  |
|  |  |  |  |  |
|  |  |  |  |  |
| **D/T**  **n=62** | Fever/chills (1,2%) | Fever/chills (6,10%) |  |  |
|  | Others (1,2%) | Skin (1,2%) |  |  |
|  |  | Musculoskeletal (1,2%) |  |  |
| **BRAFi/aPD-1**  **n=17** | Fever/chills (1,6%) | Fever/chills (3,18%) |  |  |
|  | Others (1,6%) | Skin (13,76%) |  |  |
|  |  | Musculoskeletal (6,35%) |  |  |
|  |  | Liver (1,6%) |  |  |

D/T, dabrafenib/trametinib; aPD-1, anti-program death-1 antibody; BRAFi, BRAF inhibitor; AE(s), adverse event(s).
